# 

**Published:** 1889-12

**Authors:** 


### LITERARY.

*The Century Magazine*, still holds the lead of our American Monthlies. The autobiography of Joseph Jefferson, the renowned actor, is not the least of its new offerings.

We are under obligations to the several authors and compilers for the following papers :

*Bulletin* of the *Agricultural Experiment Station* of *Cornell University*, No. X.

*Highway Improvement.* An interesting address by Col. Albert A. Pope, of Boston.

A *Plea* in favor of *Early Laparotomy*, etc., by Prof. N. Senn, M.D., Ph. D., of Milwaukee.

*Past Present and Future*, of *Woman*, by Dr. Joseph Simms, published at San Francisco, Cal.

*Death Ends All*, or the Mysteries of *Life* and *Death* naturally revealed. By Walter Hague, Philadelphia. R. Staley & Co.

*Death Not a Necessity.* A treatise on Immortality, by Joseph Wheeler, St. Louis.

---

### BRUNO.

Homage to Bruno ?—yes, his bronze shall grace
The proudest seat in Rome, and fitly so,—
There let his image sanctify the place
Where Bruno died three hundred years ago.

E'en where the mounting flames their torture lent,
And the fierce mob, without the will to save,
Looked coldly on till Bruno's life was spent,
Despoiled of country and denied a grave.

Now to the rescue come the tardy years,
With pomp and peans and the glad acclaim
Of men emancipate of slavish fears,
To wrest from malediction Bruno's name.

For Bruno dared proclaim his honest thought.
In purpose resolute, in reason free
To challenge error wheresoever taught.
For this he died ! for THIS, oh Italy.

For THIS, brave Italy, he lives to-day,
For ye have lit anew the sacred flame,
To guide a Nation on its upward way,
Divinity led, to freedom and to fame.

—LA CROIX.

Giordano Bruno, tortured and burned at the stake for his liberal opinions, in Rome A. D. 1600. The Italian government has but recently erected a statue to his honor, in the eternal city.

